# Immunological Outcomes Mediated Upon Binding of Heat Shock Proteins to Scavenger Receptors SCARF1 and LOX-1, and Endocytosis by Mononuclear Phagocytes

**DOI:** 10.3389/fimmu.2019.03035

**Published:** 2020-01-10

**Authors:** Ayesha Murshid, Thiago J. Borges, Cristina Bonorino, Benjamin J. Lang, Stuart K. Calderwood

**Affiliations:** ^1^Department of Radiation Oncology, Beth Israel Deaconess Medical Center and Harvard Medical School, Boston, MA, United States; ^2^Renal Division, Schuster Family Transplantation Research Center, Harvard Medical School and Brigham and Women's Hospital, Boston, MA, United States; ^3^Laboratório de Immunoterapia, Departmento de Ciências Básicas da Saúde, Universidade Federal de Ciências da Saúde de Porto Alegre, Porto Alegre, Brazil; ^4^Department of Surgery, School of Medicine, University of California, San Diego, San Diego, CA, United States

**Keywords:** heat shock proteins, scavenger receptor, immunity, macrophage, dendritic

## Abstract

Heat shock proteins (HSP) are a highly abundant class of molecular chaperones that can be released into the extracellular milieu and influence the immune response. HSP release can occur when cells undergo necrosis and exude their contents. However, HSPs are also secreted from intact cells, either in free form or in lipid vesicles including exosomes to react with receptors on adjacent cells. Target cells are able recognize extracellular HSPs through cell surface receptors. These include scavenger receptors (SR) such as class E member oxidized low-density lipoprotein receptor-1 (LOX-1, aka OLR1, Clec8A, and SR-E1) and scavenger receptor class F member 1 (SCARF1, aka SREC1). Both receptors are expressed by dendritic cells (DC) and macrophages. These receptors can bind HSPs coupled to client binding proteins and deliver the chaperone substrate to the pathways of antigen processing in cells. SR are able to facilitate the delivery of client proteins to the proteasome, leading to antigen processing and presentation, and stimulation of adaptive immunity. HSPs may also may be involved in innate immunity through activation of inflammatory signaling pathways in a mechanism dependent on SR and toll-like receptor 4 (TLR4) on DC and macrophages. We will discuss the pathways by which HSPs can facilitate uptake of protein antigens and the receptors that regulate the ensuing immune response.

## Introduction

Heat shock proteins (HSPs) are the major components of a primordial cellular responses to proteotoxic stress, and the resultant production of many HSP species is collectively described as the heat shock response (HSR) (1) (Table 1). Classic activators of the HSR such as heat shock, lead to rapid denaturation of intracellular proteins resulting in dysfunctional intermediate conformational states that are prone to aggregation. Cell survival necessitates an almost immediate and abundant induction of the HSPs that halt the aggregation cascade and permit refolding of cellular proteins and restoration of normal protein function (18). Beyond protein refolding, HSPs also function to facilitate trafficking of their client substrates between subcellular compartments and mediate protein-protein interactions. They are hence referred to as molecular chaperones (18, 19). The HSP family contains a number of members that belong to different protein families, but each has in common a role in stepwise protein folding (Table 1) (2, 4, 5). However, in addition to their various intracellular functions, HSPs have also been detected in the extracellular spaces and circulation. This phenomenon may be the result of the death of stressed cells and release of the highly amplified HSP cohort (6). Although HSPs lack the leader sequences required for the classical secretion pathways (20) they may be released from viable cells by non-canonical secretion pathways (21). Alternative mechanisms of HSP secretion have been described. These pathways include the secretion of free Hsp70 by a pathway similar to that utilized for non-canonical secretion of interleukin-1 beta, involving secretory lysosomes, or alternatively HSPs may be packaged in lipid vesicles known as exosomes and released to the extracellular milieu of tumors and normal tissues (7, 21–23). The extracellular HSPs may exert a number of effects on adjacent cells after their secretion, such as activation of antigen-presenting cells (APCs), including monocytes, dendritic cells (DC) and macrophages, as well as causing increased mobility and metastasis in target cells as has been observed in wound healing and cancer scenarios (3, 24–26). Hsp70 and Hsp110 have been utilized effectively in anticancer vaccines, in which they function as carriers of antigenic peptides that can be efficiently taken up and processed by APCs and presented to T lymphocytes (8, 9, 16, 17, 27–29). Understanding how HSPs are bound by acceptor cells and taken up is therefore important in determining the properties and function of extracellular HSPs.

**Table 1:** Mammalian heat shock proteins.

**HSP type**	**Intracellular role**	**Extracellular role**	**Receptor**	**References**
Hsp27	Prevents protein	Inflammatory	TLRs	(2)
	Aggregation			
Hsp60	Chaperonin	inflammatory?	TLR4?	(3)
Hsp70	Initial stages of	Inflammatory?	SR, CD91	(4–7)
	Protein folding	immune	TLR4?	(8–10)
Hsp90	Folding to the	Antigen chaperone	SR, CD91	(11–13)
	Functional state	Cell motility		(14, 15)
Hsp110	Chaperone	Antigen chaperone	SR	(16, 17)

## An Overview of HSP Receptors

**Figure 1:**
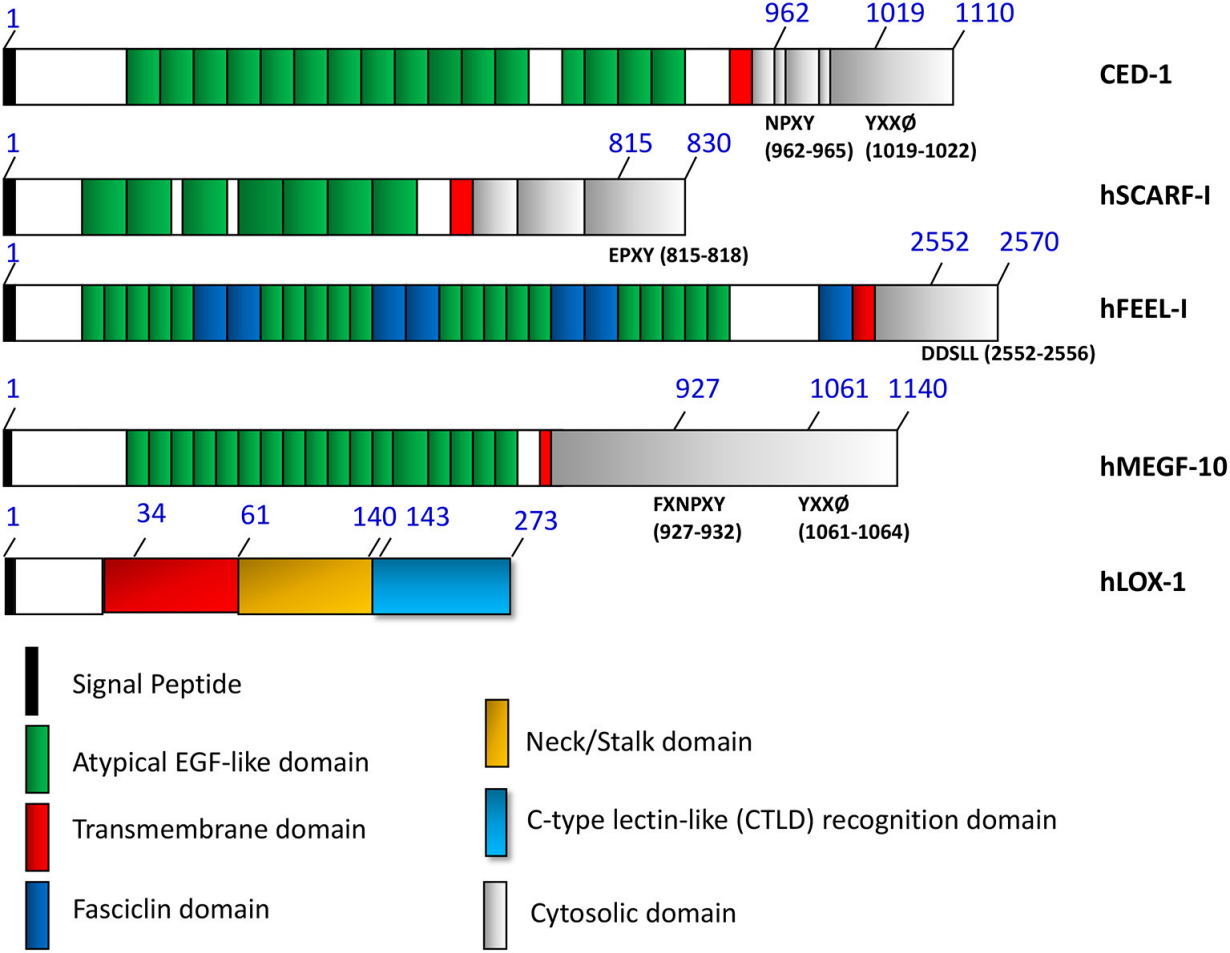
The domain structures of HSP binding and related cell surface receptors.

Most of the biological effects of extracellular HSPs identified to date have involved their binding to surface receptors on target cells prior to their internalization (10, 30). However, the entire spectrum of dedicated high affinity receptors for the HSPs have not been identified in studies carried out so far. The first protein to be identified as an HSP receptor was CD91/alpha2 macroglobulin receptor, which is a low density lipoprotein (LDL) binding protein currently known to be a highly versatile receptor for over 30 other ligands (31). This multi-subunit protein appears to be a common receptor for most of the HSPs involved in immune responses. There was some controversy originally regarding the significance of this finding, as CD91 was suggested to be the receptor involved in antigen cross-presentation by DC in response to HSP vaccines, although most types of DC do not appear to express endogenous CD91 (11, 30). However, CD91 has since been shown to be a receptor for Hsp90α in wound healing and cancer metastasis scenarios and signaling pathways downstream from the receptor appear to mediate effects of the chaperone on cell motility, a key property in wound healing and metastasis (32). The class E and F scavenger receptors LOX-1 and SCARF1 are the major receptors for HSP-peptide complexes, mediating antigen uptake and processing (10–12, 33) (Figure 1). The scavenger receptors, although not structurally related, share common functions including the binding, endocytosis and thus detoxification of oxidized LDL by vascular endothelial cells (33, 34). They are key players in the removal of oxidized LDL from the circulation and protection from the morbidity associated with atherosclerosis (35). Both LOX-1 and SREC1 are also expressed on DC and macrophages and play key roles in antigen cross-presentation mediated by HSP- peptide complexes (HSP-PC) (11, 36). In this review, we will describe the roles of these SR members in mediating extracellular HSPs-triggered responses, focusing mainly in their interaction with the Hsp90α. A really puzzling feature of this system is that most SR members are not structurally related but bind to a common ligand, while HSPs of different chaperone families often bind to the same scavenger receptor species, although also lacking structural relationship (12, 33) (Figure 1, Table 1).

It is not clear which property of HSPs prompts their binding to scavenger receptors. However, in addition to binding to oxidized LDL, members of the scavenger receptor family can bind to proteins with other modifications (acetylated LDL) as well as to polyanionic ligands such as poly-IC, findings which may cast some light on interactions with HSPs (11, 33). HSPs have been shown to be phosphorylated and acetylated, modifications that would increase their net negative charge (37, 38). Future studies would be required to clarify this issue. When LDL particles are oxidized, they assume a net negative charge and additionally phospholipid moieties are added to the LDL particle protein apolipoprotein B100 (39–41). These phospholipid residues fit into a hydrophobic tunnel formed by surface LOX-1 dimers (42). HSP binding to scavenger receptors may therefore involve the ability of the chaperones to recognize hydrophobic patches on client proteins as well as the charge interactions mentioned above (5).

## HSP Receptors and Detoxification of HSP-Peptide Complexes and Dead Cells

The primary role of the scavenger receptor family seems to be removal of oxidized LDL from the circulation (35). It is also possible that this role may be recapitulated in interactions with HSP-PC, a complex which may flood the local circulation after tissue trauma, a result of the large scale cell death by necrosis that may ensue. Such complexes may be able to prime immune and inflammatory responses in damaged tissues and it may be incumbent on mononuclear phagocytes to rapidly bind and endocytose such structures using the scavenger receptors (43, 44). It is known that LOX-1 and SCARF1 can also bind cell corpses and remove them from the extracellular spaces (45, 46). Uptake of HSP-PC may thus be part of a general detoxification process exerted by scavenger receptors, operating in damaged tissues. The scavenger receptors could also be involved in uptake and removal of HSP-containing exosomes given their abilities to bind lipid structures such as oxidized LDL and cell corpses (46). SCARF1 is a paralog of the cell corpse receptor CED-1 expressed in *C. elegans* (35, 47). In addition, more closely related paralogs of CED-1 have been unearthed and could be putative HSP receptors. These include *Drosophila* gene *draper* and the mammalian MEGF10, MEGF11, and MEGF12 (48–51). Each of these proteins contains multiple EGF-like motifs in the extracellular domain that may be recognition sequences for apoptotic bodies and play roles in dead cell clearance (Figure 1). Another protein with multiple EGF-like motifs in its extracellular domain that can bind to HSPs and apoptotic cell corpses is the Class H scavenger receptor FEEL-1/stabilin-1 (30, 33, 52) (Figure 1). Its role in responses to extracellular HSPs is currently unclear.

## Pathways of Scavenger Receptor-Mediated Endocytosis

**Figure 2:**
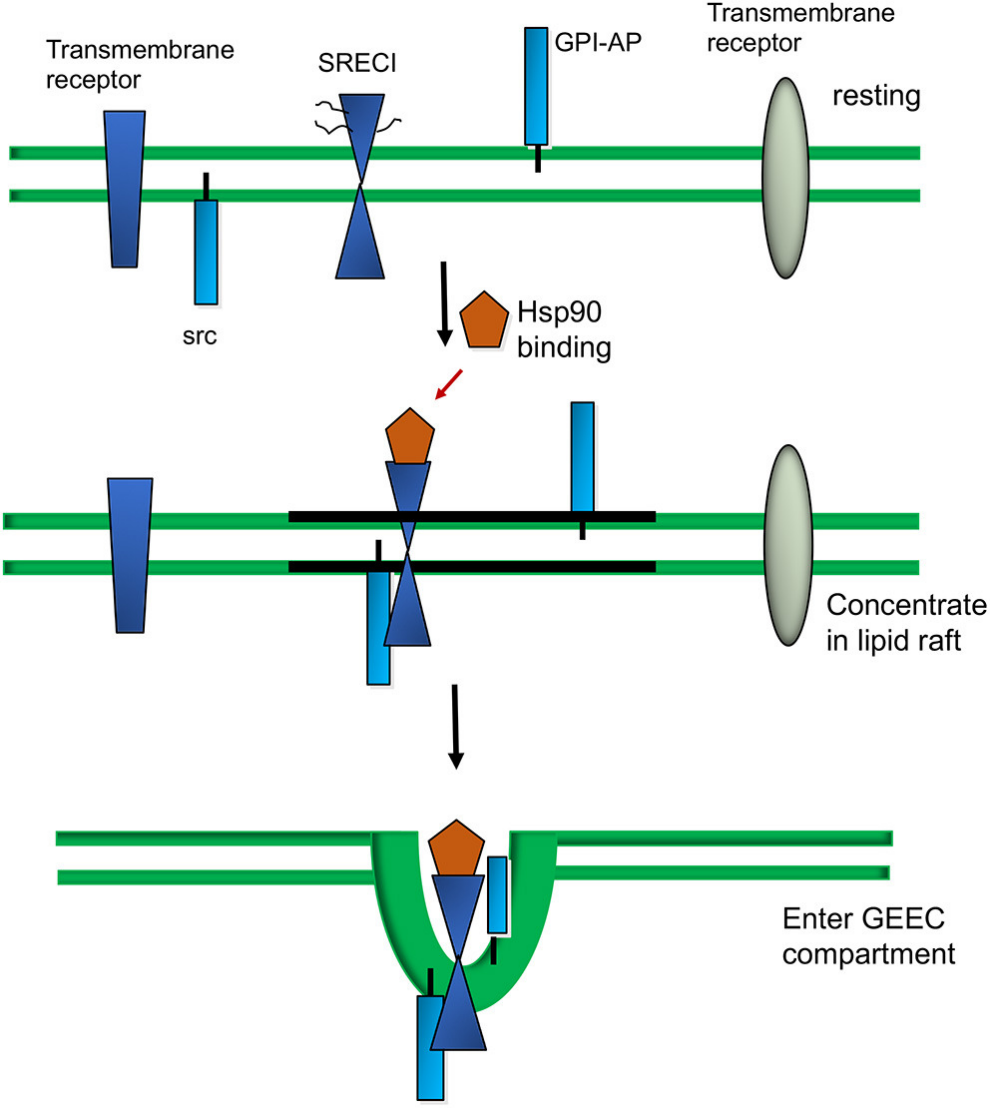
Extracellular Hsp90 -triggered sorting of SCARFI into lipid rafts and the GEEC internalization pathway.

The properties of the SR as endocytic receptors with a wide range of selectivity makes them effective intermediaries in sampling the local extracellular milieu of APC for potentially antigenic molecules. Thus, both LOX-1 and SCARF1 are expressed in DC and other mononuclear phagocytes (11, 36). There are a number of pathways by which extracellular molecules can enter cells. These include endocytosis, a process which involves the association of molecules with cell surface invaginations, uptake in an actin-dependent manner, and then fusion of the engulfed vesicles with intracellular endosomes. The major canonical pathway is clathrin-mediated endocytosis, a process that involves pit-like structures inserted into the plasma membrane which are lined with clathrin, a trimeric protein that stabilizes the pits (53). Molecules, sometimes associated with receptors, are then engulfed in clathrin coated vesicles that are found in the majority of cells. There is a second, less prevalent pathway, involving the protein caveolin found in structures known as caveolae, 50 nm invaginations that can also mediate endocytosis of extracellular molecules (54). However, both LOX-1 and SCARF1 have been shown to take up their ligands in a clathrin and dynamin-independent manner, utilizing a more unconventional endocytic pathway (36, 55). The mechanisms involved in endocytosis mediated through LOX-1 seem to be currently unclear although more information has accumulated regarding SCARF1. Upon ligand binding SCARF1 is internalized by DC via the GPI-AP (glycophosphatidylinositol-anchored proteins) enriched early endosome (GEEC) pathway (Figure 2) (56, 57). This pathway is mediated by uncoated tubular vesicular structures called clathrin independent carriers (CLICs) that mature into the early endocytic compartment (GEECs) (58, 59). The pathway is specialized for uptake of GPI-AP such as the folate receptor. Thus, uptake of Hsp90α- peptide complexes was not inhibited by antagonists of clathrin- and caveolin-dependent endocytosis, characteristic of the GEEC pathway (36). Endocytosis of Hsp90α-peptide complexes was however inhibited by blocking the activity of Rho GTPase CDC42, a protein shown to be involved in actin polymerization and uptake of GPI-AP through the GEEC pathway. SCARF1 became co-localized, after binding to Hsp90α-peptide complexes, with CD59, a marker GPI-AP protein that utilizes the GEEC pathway (36, 60). Proteins internalized through the GEEC pathway, such as GPI-AP are frequently associated within plasma membrane microdomains such as lipid rafts (61). These are regions of the membrane enriched in cholesterol and glycosphingolipids that are immiscible with the bulk membrane and appear to diffuse freely through this membrane (62, 63). SCARF1 is not a GPI-AP protein even though it has been shown to enter the GEEC pathway. However, another protein modification that may target transmembrane proteins such as SCARF1 to lipid rafts is S-acylation of cysteine residues close to the transmembrane domain with saturated palmitate residues capable of dissolving in the cholesterol and glycosphingolipid milieu that comprises the partitioned microdomains. SCARF1 contains five cysteine residues (Cys - 440, 441, 443, 444, 445) adjacent to the transmembrane domain (amino acids 422-442) (35, 62, 63). Thus, cysteine palmitoylation, and perhaps interaction with other proteins in the lipid rafts, may potentially recruit SCARF1 to this region. The nature and extent of partner proteins associated with SCARF1 in the rafts is not clear, although the receptor was shown to interact with the non-receptor tyrosine kinase c-Src (36). Although c-Src is likewise not a member of the GPI-AP family, it also associates with the rafts after S-acylation (63). Inhibition of c-Src activity prevented the cross-presentation of antigens associated with Hsp90α suggesting a key role for signaling through this tyrosine kinase in the antigen presentation pathways (36). Phosphorylation of key tyrosine residues within internalization motifs in the intracellular domain regulates the endocytosis of many receptors, although consensus sequences for internalization such as the NPXY motif found in CED-1 are not observed in the SCARF1 sequence (Figure 1) (35). The mechanism of regulation of SCARF1 endocytosis by c-Src thus remains to be defined. LOX-1 function has also been linked to its entry into lipid rafts and cholesterol lowering drugs inhibit its function (64).

## Scavenger Receptor-Mediated Access to Antigen Processing Pathways

Binding of antigenic polypeptides to HSPs in DC allows them to enter the pathway of antigen cross presentation and be processed in the cytoplasm and presented on major histocompatibility Class I (MHC I) proteins (13). Most of the antigens presented on MHC Class I proteins are derived from proteolytic processing of intracellular proteins via the classical Class I pathway (65). However, DC are specialized to take up extracellular antigens using receptors such as Fc, CLEC9A, DC-SIGN, DEC205 and mannose receptor, and thus funnel them into the Class I pathway, permitting surveillance of the extracellular spaces (66–70). HSPs can also bind external antigens and funnel them into the Class I pathway through LOX-1 and SCARF1 (36). For instance, following transit through the GEEC compartment, Hsp90α-peptide-SCARF1 complexes are translocated to early endosomes (36). In the case of full-length chaperoned proteins such as intact ovalbumin, antigen processing is carried out after its digestion in the proteasome in the cytoplasm and then antigenic peptides are taken up into MHC class I molecules by TAP (transporter associated with antigen processing) in the ER (36). This latter process clearly requires the chaperoned protein to escape the confines of the early endosome and enter the cytoplasm in order to be taken up by the proteasome. Hsp90α is known to facilitate this step as well as to maintain the client protein intact until it reaches the proteasome (71). Hsp90α-peptide complexes internalized in association with SCARF1 can also be processed within the endosome and antigens loaded onto MHC class II molecules prior to recycling to the plasma membrane (14). This is an essential step in efficient activation of T cell immunity and the activation of CD4+ T helper cells. Normally uptake of soluble antigens into the Class I and Class II pathways involves separate receptors (13). This scavenger receptor can therefore facilitate antigen processing by APC through the two major antigen presentation pathways and appears to play an integral role in the functioning of HSP based immunity (13). LOX-1 also participates in processing of HSP bound tumor antigens and approximately 50% of ovalbumin (sample antigen) cross-presentation appeared to be mediated through this receptor (36). Although HSP-PC binding to SCARF1 can facilitate antigen presentation through the MHC class I and class I pathways it is not clear whether this interaction can induce co-receptor induction in APC- a crucial step in adaptive immunity. For instance, creation of efficient anticancer vaccines employing large HSP family members has employed fusion of the HSPs to prokaryotic danger signals to boost inflammatory signaling and co-receptor induction (9). Interestingly, SCARF1 expression and Hsp70 vaccine anti-tumor activity is dependent on TLR2 and TLR4 function suggesting upregulation in inflammatory conditions (28).

## Lipid Rafts and Cell Signaling After HSP Binding to Cell Surface Receptors

**Figure 3:**
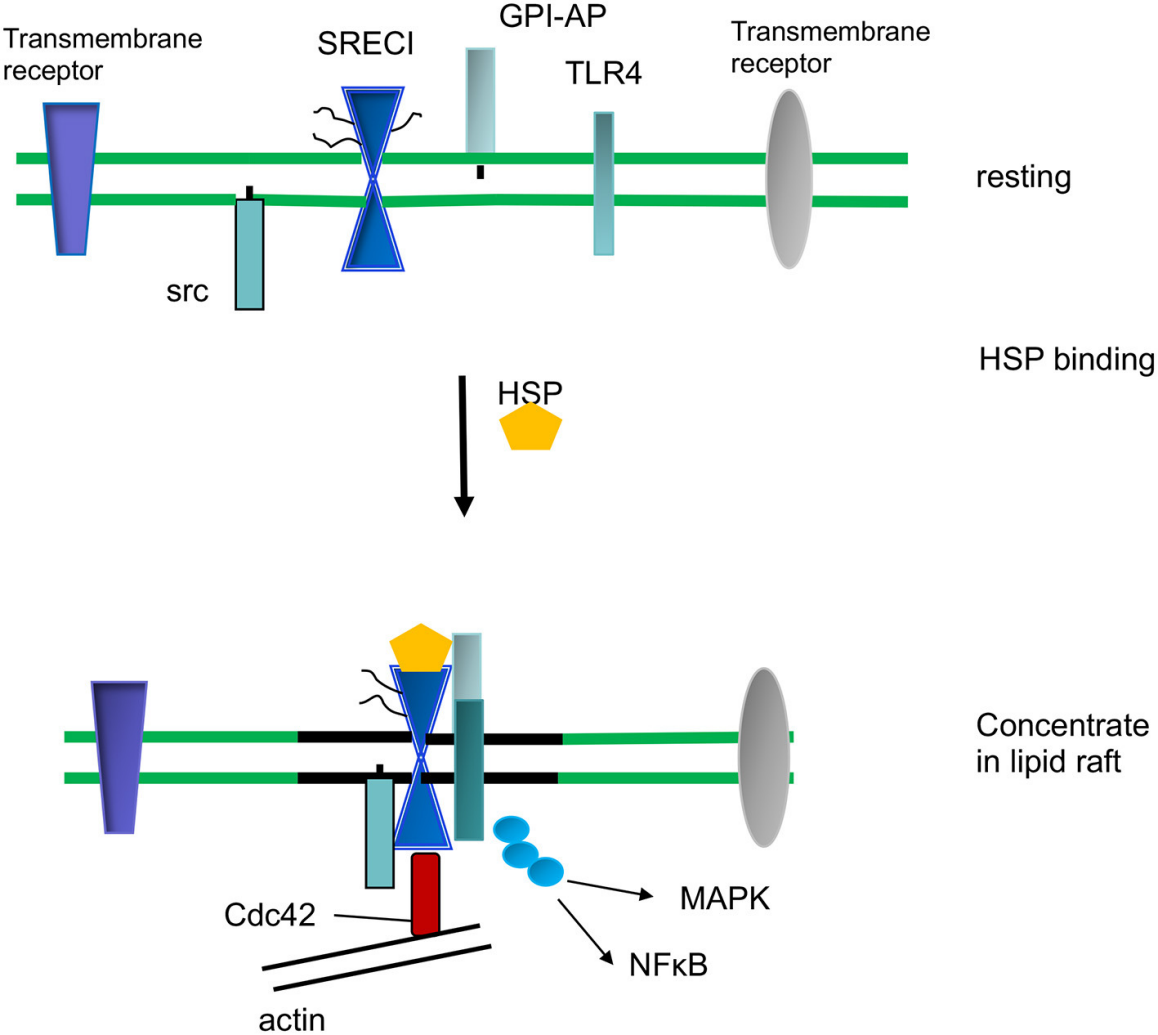
HSP-induced cell signaling and inflammation.

In addition to the import of tumor antigens by DC, HSPs may carry out key cell signaling roles within the lipid rafts of mononuclear phagocytes. The association of proteins with lipid rafts may permit them to concentrate at foci within the plasma membrane. This property depends on the ability of the rafts to diffuse within the bulk membrane and thus potentially bring together cooperating signaling molecules (62, 63, 72). As mentioned above, an example of this process is the association of SCARF1 with c-Src after Hsp90α binding, an interaction that may promote endocytosis and phagocytosis through activation of the kinase, recruitment of Cdc42 and association with the actin cytoskeleton (36) (Figure 3). SCARF1 entry into c-Src containing lipid rafts was also required for inflammatory cytokine release in mouse macrophages (73). This process involved association of SCARF1 with the pro-inflammatory Toll Like Receptor 4 (TLR4) after exposure to bacterial lipopolysaccharides (15). The association with SCARF1 in lipid rafts led to downstream signaling through TLR4, activation of the c-jun kinase, p38 MAP kinase and NF-kB signaling pathways and upregulation of interleukin 6 synthesis (73) (Figure 3). These inflammatory signaling processes required cholesterol, actin polymerization and CDC42 activity. SCARF1 and LOX-1 may also be able to recruit other signaling molecules and exposure to *outer membrane protein A* (OmpA) from *Klebsiella pneumoniae* led to recruitment of TLR2 and cytokine synthesis by the scavenger receptors (74). SCARF1 also cooperates with TLR2 in recognition of *hepatitis virus non-structural protein* by DC (75). In a similar vein, SCARF1 was shown to associate with TLR3 after exposure of macrophages to double stranded RNA and stimulate signaling through the NF-kB, MAP kinase and the IRF3 pathways (76). SCARF1 and LOX-1 may therefore play key roles in associating with cell signaling molecules and creating activating foci through the concentration of lipid rafts after binding eukaryotic or prokaryotic ligands (73, 76–78).

## SCARF1, LOX-1, and Inflammation

While it is clear that SCARF1 and LOX-1 can mediate immunity by binding HSP-associated antigens and promoting antigen cross-presentation, the effects of HSPs on inflammation are less clear (79). Discrepancies in the field were originally ascribed to the use by some investigators of purified HSPs associated with bacterial PAMPs: indeed, HSP and endotoxins undergo complex interactions that mediate inflammatory responses (80). However, Hsp70—TLR4 association and subsequent inflammatory signaling is regularly observed *in vivo* and under conditions in which endotoxin contamination of the chaperones seems unlikely [reviewed in (81)]. Nonetheless, in the case of purified Hsp90α, it was shown that while both this chaperone and LPS could bind to SCARF1 and lead the receptor to enter a lipid raft compartment, only exposure to LPS led to significant levels of pro-inflammatory signaling through this mechanism; Hsp90α alone, although entering the lipid raft compartment did not trigger inflammatory signaling (73). In addition, it has been shown that some prokaryotic HSP paralogs tend to be anti-inflammatory in contrast to the mammalian isoforms (82). The answer to this conundrum appears to be at least partially that another key class of HSP receptors is expressed on mononuclear phagocytes—*the sialic acid-binding immunoglobulin-type lectins* (Siglecs) (83, 84). The Siglec family of receptors bind to self-sialic acid residues either *in cis* or *in trans* and this interaction leads to suppression of inflammatory responses in mononuclear phagocytes (83). Upon binding to the antigen, the intracellular regions of the Siglecs become activated by phosphorylation of immunosuppressive ITIM (immunoreceptor tyrosine-based inhibition motif) domains that associate with phosphatases Shp-1 and Shp-2, leading to immune suppression (85). Hsp70 has been shown to bind to Siglecs after tissue damage suggesting a mechanisms for the immunosuppressive roles of the chaperone (86). A further complication to this scenario is that human cells can express a receptor pair SIGLEC-5 and SIGLEC-14 that can contain similar ligand binding domains and either ITIM or ITAM (immunoreceptor tyrosine-based activation motif) sequences and thus, dependent on context, are either immunosuppressive (SIGLEC-5) or immunostimulatory (SIGLEC-14) (87). Clearly further studies are essential to clarify the nature of the signaling complexes on mononuclear phagocytes that determine response to HSPs in terms of both endocytosis and inflammatory cell signaling.

## Conclusions

- The scavenger receptors LOX-1 and SCARF1 mediate binding and endocytosis of HSPs such as Hsp70, Hsp90, and Hsp110. The HSPs are taken up by a clathrin-independent mechanism involving the GEEC pathway. At least in the case of SCARF1, endocytosis requires the activity of the c-Src kinase which can bind to the receptor in lipid raft microdomains.
- Uptake by scavenger receptors may be a component of the detoxification pathways, with the effect of removing inflammatory and immune-stimulatory HSP-peptide complexes, particularly in the context of tissue injury. This process may also be beneficial in the activities of molecular chaperone-based vaccines in which the HSPs enhance antigen uptake, integrity and cross-presentation to CD8+ T lymphocytes.
- The scavenger receptors localize to lipid raft microdomains on the mononuclear phagocyte cell surface after HSP binding. This process may facilitate endocytosis through the GEEC pathway by bringing the SR in close proximity to c-Src and CDC42. In addition, concentration of scavenger receptors in lipid rafts with TLR4 and other regulatory proteins may trigger inflammatory signaling and cytokine synthesis after HSP binding.

Three HSP binding receptors SCARF1/SREC-I, FEEL-1, and LOX-1 are shown as well as related proteins. Locations of atypical EGF-like domains are indicated in—CED-1, hSCARF1, hFEEL-I/Stabilin-1 and MEGF10. Each share EGF-like consensus repeats in the extracellular domains. Tyrosine-based sorting signals are known to interact with the phospho-tyrosine domain of clathrin adaptors (NPXY for CED-1, FXNPXY and YXXØ for hMEGF10) are shown in the figure. SCARF1 does not contain these motifs and is not internalized through clathrin-mediated endocytosis. FEEL-1 is expressed mainly in intracellular compartments. A dileucine based (DXXLL for hFEEL-1) sorting signal is present in the cytosolic tails of hFEEL-1 and can also be found in mannose 6-phosphate receptors that mediate sorting between trans-Golgi network (TGN) and endosomes. LOX-1, although sharing many properties with SCARF1, including HSP binding and internalization, does not contain EGF-like motifs in its extracellular domain extracellular domain and belongs to the C-type lectin family.

Under resting conditions, SCARF1 is shown in the bulk membrane domain containing a range of surface proteins which are either transmembrane proteins such as SCARF1, GPI-AP proteins or proteins anchored to the inside of the membrane such as c-Src. Upon Hsp90α binding, SCARF1 becomes localized into lipid raft domains and co-localized with c-Src. Within 5 min of ligand binding, Hsp90α–SCARF1 complexes enter the GEEC compartment and are internalized (36). We also show proteins that remain in the bulk membrane and are not internalized through the GEEC pathway.

We show ligand (HSP) binding by SCARF1 leading to its recruitment to lipid raft microdomains in the plasma membrane. SCARF1 then coordinates interaction of c-Src, CDC42 and TLR4 and signaling through the NF-kB and MAP kinase pathways upstream of inflammatory cytokine expression.

## Author Contributions

All authors listed have made a substantial, direct and intellectual contribution to the work, and approved it for publication.

### Conflict of Interest

The authors declare that the research was conducted in the absence of any commercial or financial relationships that could be construed as a potential conflict of interest.
